# CD155/PVR plays a key role in cell motility during tumor cell invasion and migration

**DOI:** 10.1186/1471-2407-4-73

**Published:** 2004-10-07

**Authors:** Kevin E Sloan, Brenda K Eustace, Jean K Stewart, Carol Zehetmeier, Claudia Torella, Marina Simeone, Jennifer E Roy, Christine Unger, David N Louis, Leodevico L Ilag, Daniel G Jay

**Affiliations:** 1 Department of Physiology, Tufts University School of Medicine, Boston, MA, USA; 2 Xerion Pharmaceuticals, AG, Munich, Germany; 3 Department of Pathology, Massachusetts General Hospital and Harvard Medical School, Boston, MA, USA

## Abstract

**Background:**

Invasion is an important early step of cancer metastasis that is not well understood. Developing therapeutics to limit metastasis requires the identification and validation of candidate proteins necessary for invasion and migration.

**Methods:**

We developed a functional proteomic screen to identify mediators of tumor cell invasion. This screen couples Fluorophore Assisted Light Inactivation (FALI) to a scFv antibody library to systematically inactivate surface proteins expressed by human fibrosarcoma cells followed by a high-throughput assessment of transwell invasion.

**Results:**

Using this screen, we have identified CD155 (the poliovirus receptor) as a mediator of tumor cell invasion through its role in migration. Knockdown of CD155 by FALI or by RNAi resulted in a significant decrease in transwell migration of HT1080 fibrosarcoma cells towards a serum chemoattractant. CD155 was found to be highly expressed in multiple cancer cell lines and primary tumors including glioblastoma (GBM). Knockdown of CD155 also decreased migration of U87MG GBM cells. CD155 is recruited to the leading edge of migrating cells where it colocalizes with actin and αv-integrin, known mediators of motility and adhesion. Knockdown of CD155 also altered cellular morphology, resulting in cells that were larger and more elongated than controls when plated on a Matrigel substrate.

**Conclusion:**

These results implicate a role for CD155 in mediating tumor cell invasion and migration and suggest that CD155 may contribute to tumorigenesis.

## Background

Metastasis is responsible for greater than 90% of cancer-related deaths [1]. It is therefore of great importance to develop therapies that limit this process. Cell migration plays a key role in invasion, an early step in metastasis, and proteins that regulate migration are often upregulated in tumor cells [2]. Cell migration also plays a key role in the dispersal of tumor cells within a tissue. In glioblastoma, the most aggressive form of brain cancer, tumor cells disperse so extensively that common treatment approaches such as resection or radiation therapy are not effective in checking progression [3].

Both invasion and dispersal are complex processes that require migration of individual cells from the tumor core into surrounding tissue and the extracellular matrix. Cell migration requires a coordinated orchestration of complex events including polarization, protrusion, adhesion, de-adhesion, and retraction [4]. Many cell-surface proteins are involved in regulating migration. Growth factor receptors receive environmental cues and initiate signaling cascades resulting in polarization and directional migration [5]. Cell adhesion molecules (CAMs) such as integrins, cadherins, and immunoglobulin family proteins, mediate adhesion and deadhesion between a cell and its neighbors or the extracellular matrix (ECM) and can also contribute to polarization and directional motility in response to soluble ECM proteins. CAMs sit at the top of many signaling cascades that regulate actin and microtubule dynamics through Rho family GTPases [2]. While many proteins have been described to play a role in cell migration, the mechanisms through which they act remain unclear [2]. Establishing which of these proteins are required for tumor invasion and migration is an important first step to developing therapeutics aimed at limiting the metastasis or dispersal of tumor cells.

In the post-genome era, global strategies are being developed to identify new players in complex biological processes such as tumor cell invasion. Microarray experiments have identified many differentially expressed genes that may contribute to enhanced invasion [6-10], but these correlative expression changes can only suggest functional importance. RNAi screens for cancer-relevant phenotypes have already identified several new gene targets [11,12]. Such approaches are limited, however, by the availability of genome-wide RNAi libraries, the possibility of genetic compensation following chronic inactivation, and the inability of RNAi to knockdown stable proteins with slow turnover. Proteomic screens have the advantage of being able to assess the proteome in high throughput but do not directly address function [13].

To complement these approaches, we have developed a high-throughput, acute protein inactivation strategy called fluorophore-assisted light inactivation (FALI) that allows for direct assessment of protein function through the systematic inactivation of proteins [14-16]. We have previously applied this approach on a smaller scale to identify surface proteins important for tumor cell invasion and have established a role for extracellular Hsp90 in activating metalloproteinases required for invasion [16]. Here we used a larger, unbiased functional proteomic screen to demonstrate that CD155, the receptor for poliovirus, contributes to tumor cell invasion by regulating cell migration.

## Methods

### Cells

HT1080 human fibrosarcoma, Hs27 human fibroblasts, and U87MG human glioblastoma cells were obtained from ATCC. Normal human astrocytes were obtained from Cambrex Bioproducts. Cells were maintained in DMEM supplemented with 10% fetal bovine serum and penicillin/streptomycin (Invitrogen; HyClone). HT1080 cells were additionally supplemented with 0.1 mM non-essential amino acids (Invitrogen). All cells were grown at 37°C under a humidified 7% CO_2 _atmosphere.

### Antibodies

Monoclonal antibodies used for FALI were as follows: β1-integrin (JB1, Chemicon); CD155 (D171, Neomarkers; pv404.19, Beckman Coulter). Two additional CD155-specific monoclonal antibodies (5D11, ID8) were generated by fusing specific binding scFvs selected from our library to a human IgG backbone. ScFv 1A2 was used as a negative control in the migration assay. 1A2 was selected for binding to the surface of HT1080 cells (see scFv Library Generation) and was confirmed to bind to the surface of HT1080 and U87MG cells by immunocytochemistry, though its protein target is not known. Antibodies used for immunocytochemistry and immunoblotting were as follows: CD155 (D171; CD155 rabbit polyclonal, gift of Dr. Eckard Wimmer); αv-integrin (AB1930, Chemicon); ErbB2 (06-562, Upstate). Actin was visualized with rhodamine-phalloidin (Molecular Probes). Fluorophore-labeled secondary antibodies were obtained from Molecular Probes; peroxidase-labeled secondary antibodies were from Cell Signaling.

### scFv Library Generation

Spleen RNA was harvested from HT1080 immunized mice as described [16]. Immunoglobulin cDNA was synthesized using a primer mix and variable regions amplified using specific primers. Products were cloned into the phage display vector pXP10 and transfected into *E. coli *TG-1 resulting in a library of 10^7 ^independent clones. HTl080-specific scFvs were selected by immunopanning phage against fixed HT1080 lysate, resulting in 2760 binders. These scFvs were recloned into expression vector pXP7 (containing his- and E-tags) and expressed in TG-1 cells. Bacterial lysates containing scFv were prepared and tested for binding to the surface of fixed HT1080 cells by ELISA. This additional round of selection yielded 595 HT1080 surface binders. These scFvs were confirmed to bind to the surface of HT1080 by immunocytochemistry on live cells. For FALI/screening, his-tagged scFvs were purified from bacterial lysates using Ni-NTA Superflow resin (Qiagen).

### FALI

Antibodies or scFvs were conjugated with FITC (Molecular Probes) at pH 9.5 at room temperature as previously described [17]. Cells were detached with Versene (Invitrogen) and resuspended in serum-free, phenol red-free HBSS (Invitrogen). Cells were incubated with FITC-conjugated antibodies for 1 h at room temperature with gentle rocking and were then transferred in at least sextuplet to replicate clear, flat-bottomed 96-well plates on ice. One plate was illuminated for 1 h with 300 W (1 × 10^5 ^lux) blue-filtered light (Brilliant Blue #69, Roscolux) using a high-powered slide projector (Ektagraphic III, Kodak). A replicate control plate was kept in the dark for 1 h.

### Invasion and migration assays

FALI invasion assays with HT1080 cells were done as described previously [14,16] but used the scFv library described above. 1 × 10^5 ^cells were labeled with cell tracker orange, incubated with 20 μg/ml of FITC-labeled scFv, irradiated (or kept in the dark) with blue-filtered light, and 5 × l0^4 ^cells were loaded onto matrigel-coated (4 μg, top) polycarbonate membranes (8 μM, 96-well, Neuroprobe). Each sample was assayed in triplicate in at least two independent experiments. scFvs that showed a significant change in invasion (p < 0.01 and change > 2 standard deviations) were assayed a third time with a new scFv preparation. U87MG and HT1080 migration assays were performed essentially as above, but excluded matrigel and used 8μM, 96-well Fluoroblok membranes (BD Biosciences). Cell invasion or migration was quantified using a fluorescent plate reader (SpectraFluor Plus, Tecan) and confirmed by visualization under an inverted fluorescent microscope.

### Immunoprecipitation and Mass Spectrometry

To identify scFv protein targets, candidate scFv genes were re-cloned into expression vector pXP14, containing a strep-tag. Purified scFvs were coupled to StrepTactin Sepharose (50 μg/50 μl resin) and the washed scFv-beads were added to 1 mg HT1080 lysate. The scFv-target complexes were eluted (10 mM D-desthiobiotin, 0.1% Tween 20 in PBS) and the immunoprecipitated proteins were analyzed by SDS-PAGE and silver staining. In a parallel experiment, the immunoprecipitated proteins were subjected to deglycosylation using N-glycosidase F prior to SDS-PAGE analysis. Stained bands were excised and subjected to in-gel tryptic digestion. The peptide fragments were extracted from the gel, desalted on ZipTip μC18, the eluted peptides spotted on a Teflon-coated MALDI target, let dry and overlayed with 1 μl of a 3.5 mg/ml solution of α-Cyano-hydroxycinnamic acid. The samples were analyzed on a STR-DE Voyager MALDI mass spectrometer (Applied Biosystems) and the obtained peptide masses were used for protein identification via peptide mass fingerprint, searching all entries for the species *Homo sapiens *in the NCBI and SwissProt databases. Alternatively, the extracted peptide fragments were analyzed by nano electrospray mass spectrometry (nanoES-MS) on a Q-STAR QqTOF mass spectrometer (PE SCIEX). Relevant ions were selected for CID (collision induced dissociation)-MS and the obtained fragment ion data used for Peptide Sequence Tag database search.

### CD155 siRNA

A double stranded siRNA oligonucleotide targeting CD155 (5'-CAACUUUAAUCUGCAACGUdTdT-3') was chemically synthesized (Dharmacon Research) and transfected into HT1080 cells using Oligofectamine (Invitrogen) following manufacturers instructions using 200 nM siRNA per 10 cm dish. Cells were incubated with siRNA in OptiMEM (Invitrogen) for 6 hrs after which time normal growth media was added. Cells were then incubated for 48–72 h to achieve >80% knockdown of CD155. Control cells were transfected with a scrambled siRNA oligonucleotide at matching concentration. Cells were then inserted into the migration assay described above or used for morphology experiments.

### Cell morphology measurements

HT1080 cells were co-transfected with siRNA (scramble control or CD155-specific) and fluorescently labeled oligonucleotide (10 nM, Sequitur) for 48 hours. Cells were detached with Versene, and 5–15 × 10^3 ^cells were loaded onto glass coverslips coated with Matrigel (2μg/ml, BD Biosciences). After 2 hours of incubation at 37°C/7% CO_2_, cells were fixed and immunostained for CD155 using mAb D171. Cells were visualized with a Nikon Diaphot 200 microscope and images taken of >100 transfected cells (containing fluorescent oligo). Analysis of images was performed with OpenLab software (Improvision). Measurements of cell area, perimeter, and shape were made for each condition.

### Wound healing assay

8-chamber slides (Falcon) were coated with matrigel (2 μg/ml) and blocked with FCS. 100,000 cells were plated in each well and grown to confluency. A 20-gauge needle was used to create a linear wound and cells allowed to recover for 3 h at 37°C. Cells were fixed and processed for immunocytochemistry as described below.

### Immunocytochemistry

Cells were fixed in PBS/4% paraformaldehyde/4% sucrose, blocked in PBS/0.01% triton-x-100/10%FBS, and incubated in primary antibody for 1 h at rt. Appropriate species-specific secondary antibodies conjugated to Alexa 488 or 594 were used to visualize antibody staining on a Leica SP2 confocal microscope using LCS software. Antibodies: CD155 (D171), αv-integrin (AB1930), actin (rhodamine-phalloidin, Molecular Probes), ErbB2 (6562). Secondary antibodies alone were used to control for non-specific staining. For primary tissue, paraffin sections from a human tissue library were stained with biotinylated anti-CD155 human mAb 1D8 at 200 μg/ml and visualized with streptavidin-hrp and NovaRed substrate. For glioblastoma tumors, we used tissue microarrays, made from 20 primary tumors arrayed in quadruplicates of 1 mm cores with a Beecher Instrument arrayer. Positive staining was visualized with DAB substrate.

### Immunoblotting

Pelleted and PBS-washed cells were lysed in NP40 lysis buffer (0.2% NP-40, 150 mM NaCl, 20 mM Tris pH7.5, 10% glycerol) with protease inhibitors (Roche) at 4°C. Lysates were cleared by centrifugation and quantified using the DC Protein Assay (BioRad). 30 μg lysate was separated on a 10% SDS-PAGE gel and transferred to a nitrocellulose membrane. Membranes were blocked in 5% nonfat dry milk in PBS and probed with primary antibody overnight. Antibody binding was detected with peroxidase-conjugated secondary antibodies (Cell Signaling) and visualized using ECL substrate (PerkinElmer).

## Results

### Functional proteomic screen reveals a role for CD155/PVR in tumor cell invasion and migration

**Figure 1:**
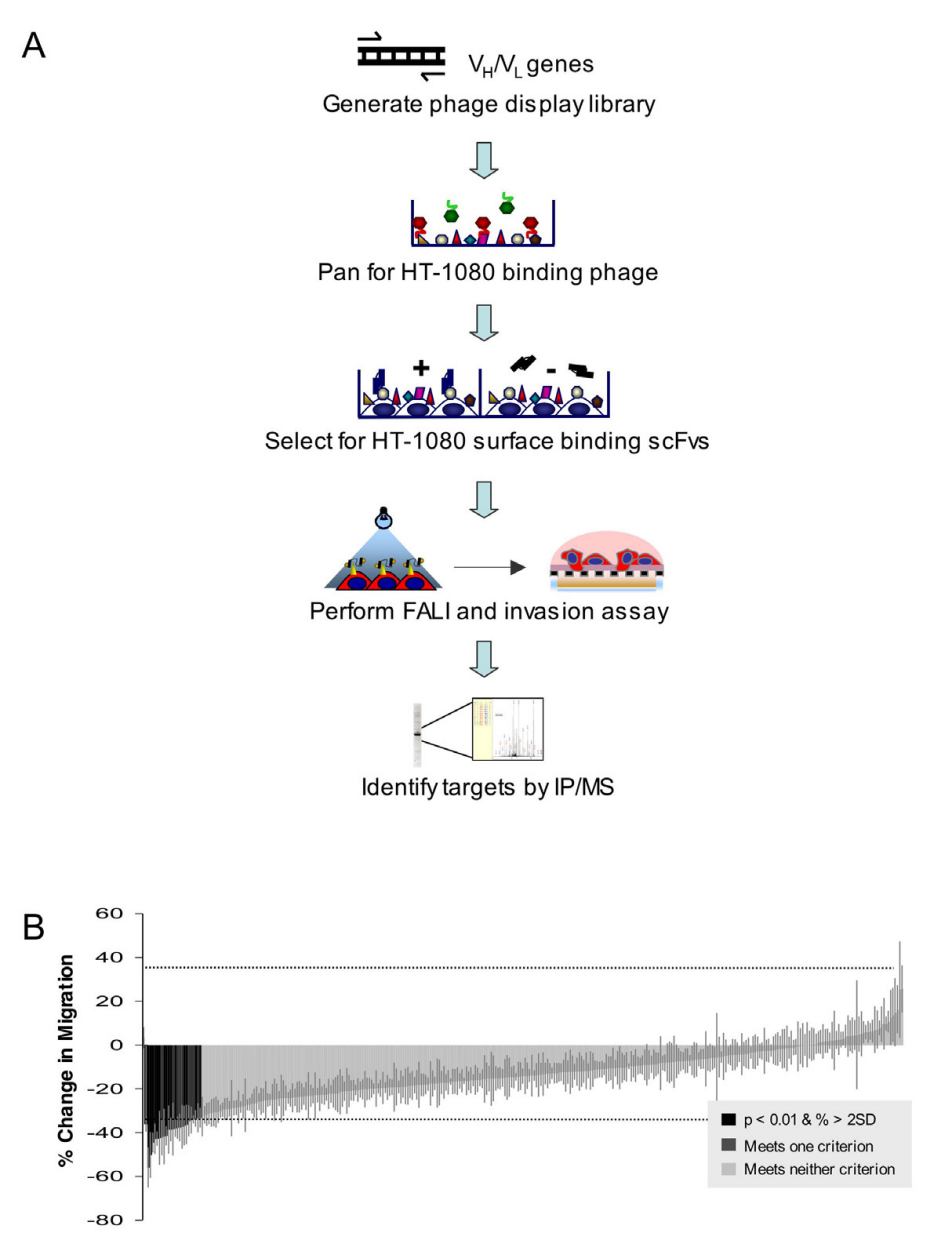
**Proteomic screen reveals a role for CD155 in tumor cell invasion. (a) **Mice were inoculated with fixed HT1080 cells. A phage display library was generated from immunoglobulin genes (V_H _and V_L_) to generate recombinant scFvs (10^7 ^phage). Phage were immunopanned against HT1080 cells, and 2,760 binders were isolated. 595 of the resulting scFvs were further selected as HT1080 surface binders. These surface binders were conjugated to FITC and used to inactivate their protein targets by fluorophore-assisted light inactivation (FALI). The invasiveness of FALI- and control-treated cells was then compared using a high-throughput transwell assay in which cells were challenged to invade through a matrigel-coated 8βM filter towards a serum chemoattactant. Protein targets of candidate scFvs were identified by immunoprecipitation and mass spectrometry. **(b) **338 scFvs were screened in triplicate in two independent experiments using the FALI-invasion assay. 6 scFvs caused a significant reduction of HT1080 invasion compared to non-FALI controls (p < 0.01, unpaired t-test and an amplitude change of greater than twice the average standard deviation).

We generated a recombinant single chain variable fragment (scFv) antibody library that recognized proteins on the surface of HT1080 fibrosarcoma cells, a highly invasive tumor cell line. ScFvs were selected from a phage display library generated from mice immunized with fixed HT1080 cells [16]. Genes encoding the selected scFvs were re-expressed as scFv antibodies and selected by ELISA for HT1080 surface binding. The selected scFvs were conjugated to fluorescein and used to acutely inactivate their protein targets by fluorophore-assisted light inactivation (FALI). The invasiveness of FALI- and control-treated cells was then compared using a 96-well transwell assay incorporating a matrigel-coated 8μM filter above a serum chemoattactant (Fig. 1a). 338 scFvs were screened in triplicate using this FALI-invasion assay. 15 scFvs caused a significant reduction of invasion compared to non-FALI controls (p < 0.01, unpaired t-test) and an amplitude change of greater than twice the average standard deviation. The majority of scFvs screened (323) had no significant effect on invasion and serve as an internal control. The 15 positive scFvs were re-screened using fresh scFv preparations in triplicate, and six scFvs were selected that continued to satisfy both criteria of significance (Fig. 1b). Sequencing of the scFv cDNAs revealed two unique groups of clones. The first group (I) contained a single scFv binder while the second group (II) included 5 scFvs with identical or nearly identical variable heavy and light chains. Protein targets of the two scFv groups were immunoprecipitated and identified by mass spectrometry.

**Figure 2:**
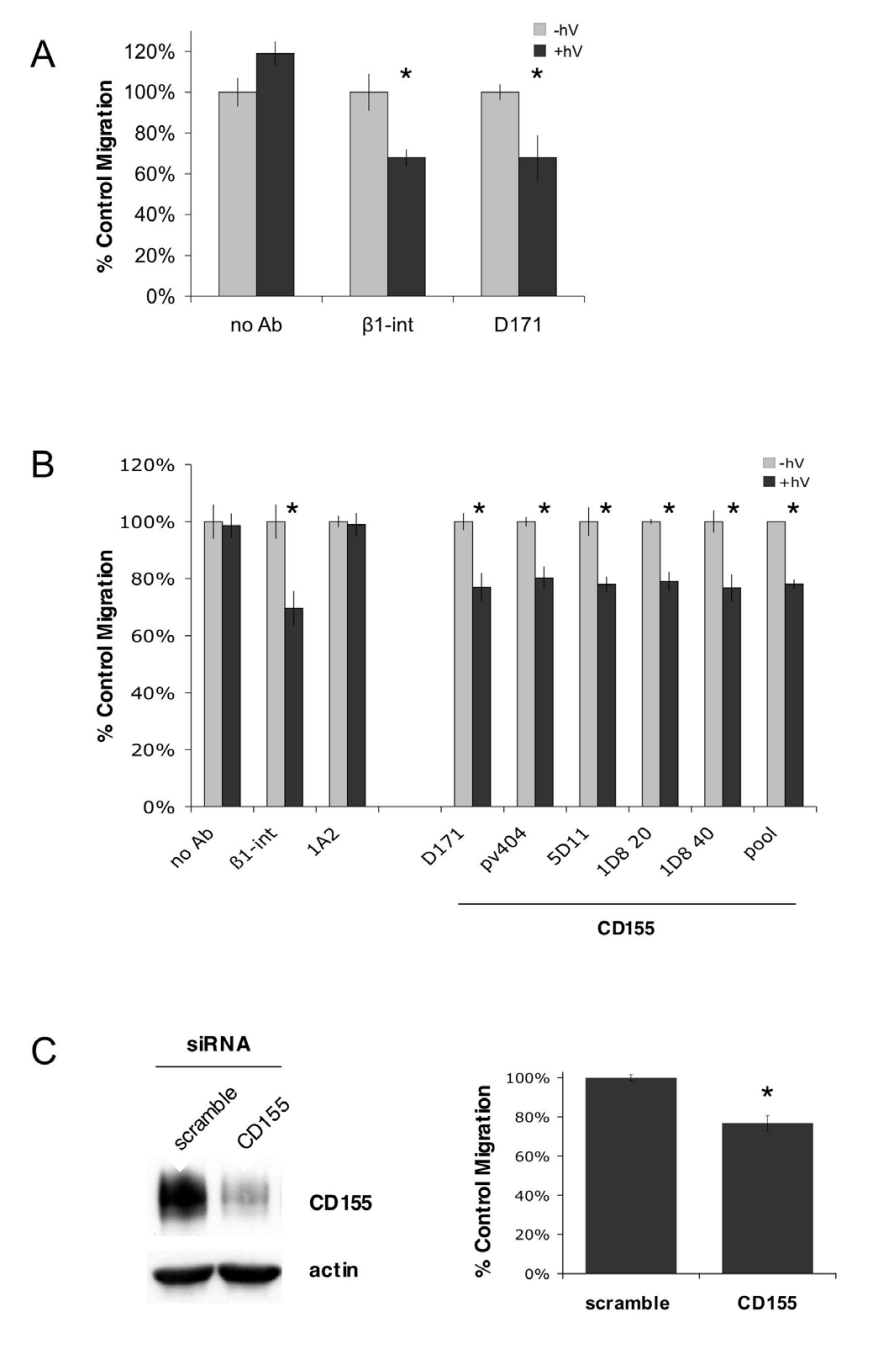
**CD155 mediates tumor cell invasion and migration. (a) **FALI of CD155 resulted in a significant inhibition of HT1080 transwell invasion through matrigel towards a serum chemoattractant (Δ *invasion ± FALI: no Ab 19 ± 6%; β1-int 32 ± 4%; CD155 32 ± 11%)*. **(b) **FALI of CD155 using multiple mAbs resulted in a significant inhibition of HT1080 transwell migration towards a serum chemoattractant (Δ *migration ± FALI: no Ab l ± 4%; β1-int 30 ± 6%; 1A2 (control, surface binding scFv) 1 ± 3%; CD155: D171 23 ± 4%; pv404 20 ± 3%; 5D11 22 ± 4%; 1D8(20) 21± 2%; 1D8(40) 23 ± 4%; CD155pool 22 ± 1%)*. FALI with CD155 mAb pools did not yield greater inhibition, suggesting maximal inactivation or that the binders share a common epitope. **(c) **CD155-specific siRNA duplexes were used to knockdown protein expression by about 90% by 72 h. Knockdown of CD155 in HT1080 cells resulted in a significant decrease in transwell migration compared to control cells transfected with a scrambled duplex *(23 ± 4%)*. * *indicates p < 0.01, t-test.*

The target of group I was α3βl-integrin, a promiscuous extracellular matrix binder that plays multiple roles in cell adhesion, morphology, migration, and invasion [18]. Identifying α3βl-integrin demonstrates the ability of our screen to reveal proteins with a confirmed role in tumor invasion. The target of group II was CD155, the poliovirus receptor. CD155 is a member of the nectin subclass of immunoglobulin domain proteins whose cellular function has not yet been established. To confirm the identification of CD155 as a mediator of invasion, we repeated the invasion assay using a previously characterized monoclonal antibody specific for CD155 (D171). FALI of CD155 significantly inhibited invasion of HT1080 cells by 32% (p < 0.01, t-test; Fig. 2a), consistent with the screening result.

In order to determine whether this result was specific to invasion through matrigel or due to effects on cell migration, we repeated the transwell experiment in the absence of matrigel using several different CD155-specific monoclonal antibodies, either previously characterized (D171, pv404) or newly generated by fusing the variable regions from our CD155-binding scFvs to a human IgG backbone (5D11, 1D8). FALI of CD155 significantly inhibited transwell migration by 20 to 23% (p < 0.01, t-test; Fig. 2b), suggesting a role for CD155 in cell migration that is responsible for the bulk of its contribution to invasion. FALI in the absence of antibody or in the presence of scFv 1A2, which had no effect in the invasion screen, did not alter migration. FALI of CD155 did not affect cellular viability or proliferation (data not shown). FALI with combinations of CD155 binders did not enhance the inhibition of migration (Fig. 2b), suggesting that inactivation was maximal or that the antibodies bound and saturated a common epitope.

To further validate the role of CD155 in tumor cell migration, we developed a siRNA duplex targeting CD155 mRNA as a complementary, chronic means of protein inactivation. Knockdown of CD155 mRNA yielded ~90% depletion of CD155 protein at 72 h and a 23% reduction in migration compared to control cells transfected with a scrambled siRNA duplex (p < 0.01, t-test; Fig. 2c). The extent of migration inhibition due to siRNA was equal to the inhibition seen by FALI, supporting the specificity of the antibodies. The observed changes in migration were not due to changes in survival or proliferation as measured by an MTS viability assay (data not shown). Taken together, the results from FALI and siRNA knockdown of CD155 clearly establish a role for CD155 in tumor cell invasion and migration.

### CD155/PVR protein is highly expressed in cancer cells and primary tumors compared to normal counterparts

Since our screen was performed using a library selected for binding to HT1080 surface proteins, and thus might have a tendency to target proteins upregulated in these cells, we profiled CD155 expression in these and other cells. Lysates prepared from normal fibroblasts, fibrosarcoma, normal astrocytes, and GBM cells were tested for expression of CD155 by immunoblot using a polyclonal CD155 antibody (gift of Eckard Wimmer).

**Figure 3:**
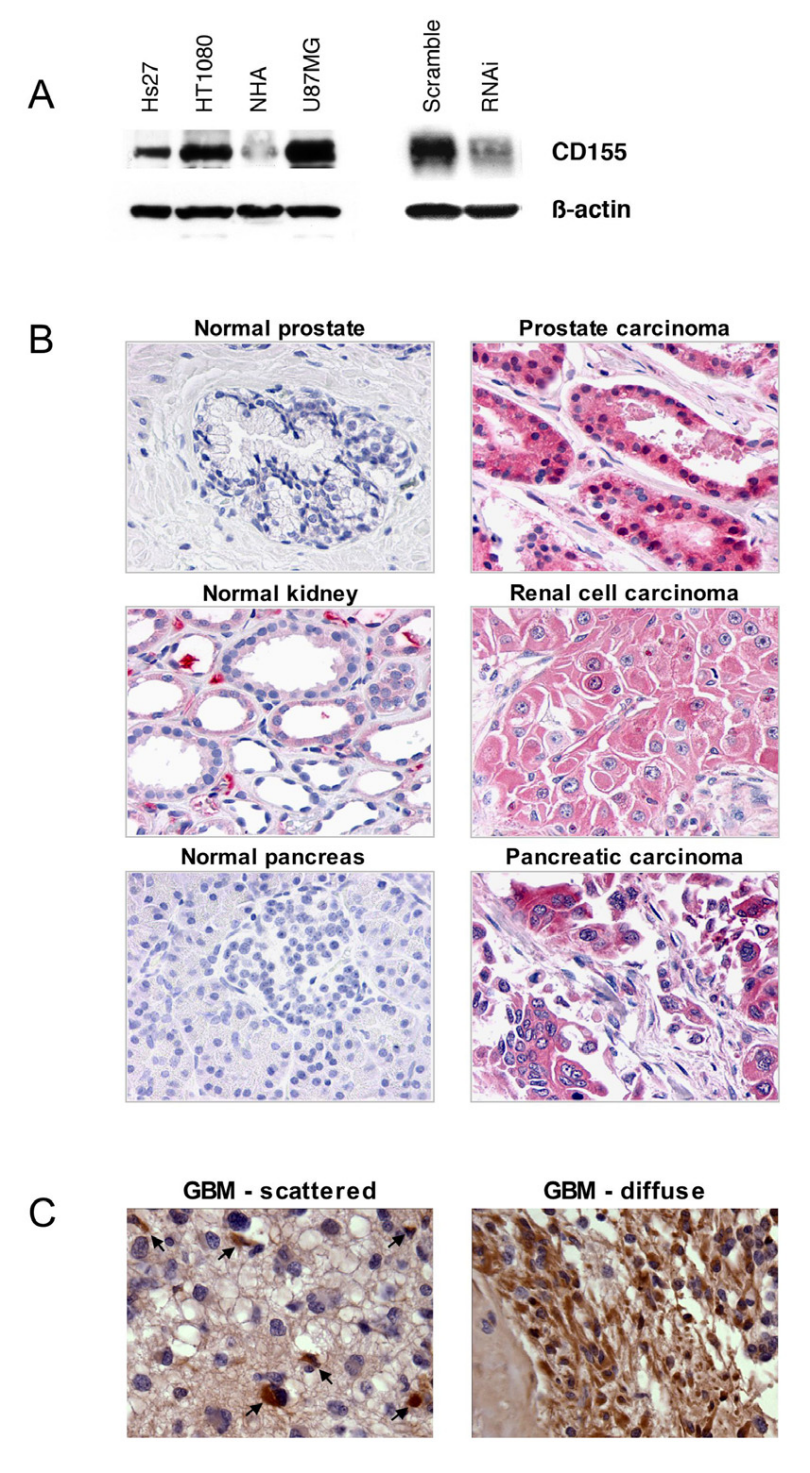
**CD155 is highly expressed in cancer cells and primary tumors. (a) **Lysates from normal and cancer-derived cell lines were immunoblotted for CD155. CD155 expression was elevated in fibrosarcoma cells (HT1080) vs. normal fibroblasts (Hs27) and also in glioblastoma cells (U87MG) vs. normal human astrocytes (NHA). Antibody specificity is demonstrated using HT1080 cells in which CD155 expression is knocked down by RNAi. β-actin is shown as a loading control. **(b) **CD155 protein expression was examined by immunocytochemistry across a panel of normal and cancer tissue samples. In normal tissue we observed moderate staining in kidney, plasma cells, liver, lung, theca interna of the ovary, and testis. No staining was observed in normal adrenal, bladder, brain, breast, colon, heart, pancreas, placenta, prostate, skin, skeletal muscle, small intestine epithelium, spleen, stomach, thymus, thyroid, or uterus. In cancer tissue, significant staining was observed in prostate, renal cell, and pancreatic carcinomas as well as in colon, non-small cell lung, ovarian, and breast carcinomas. **(c) **High CD155 protein expression was also observed in glioblastoma tumor tissue. Two types of staining were evident: scattered positive cells (arrows) in a primarily negative sample, or diffuse staining across many cells in a sample.

High levels of CD155 expression were observed in HT1080 and U87MG cells whereas the protein was only weakly expressed in their non-tumor counterparts (Fig. 3a). Since HT1080 and U87MG cells are highly invasive while hs27 cells and normal astrocytes are not (personal observation), these results suggest that upregulation of CD155 may contribute to an invasive phenotype.

Given our finding that CD155 appeared to be overexpressed in our model tumor cell lines, we evaluated CD155 expression levels in normal and cancerous human tissue. We performed immunohistochemistry on paraffin sections taken from a tissue library using mAb 1D8. In normal tissue we observed moderate staining in kidney, plasma cells, liver, lung, theca interna of the ovary, and testis (data not shown). No staining was observed in normal adrenal, bladder, brain, breast, colon, heart, pancreas, placenta, prostate, skin, skeletal muscle, small intestine epithelium, spleen, stomach, thymus, thyroid, or uterus (at least two samples examined per tissue). In cancer tissue, we observed extensive staining in a subset of samples taken from several different tumor types (Fig. 3b). These included prostate carcinoma (4 out of 10 samples examined), renal cell carcinoma (4/10), pancreatic carcinoma (7/10), colon carcinoma (2/10), ovarian carcinoma (2/10), non-small cell lung carcinoma (1/10), and breast carcinoma (1/10). Since CD155 had previously been suggested to be upregulated in glioblastoma (GBM) tumors [19], we performed immunostaining on a tissue array to examine CD155 protein expression across twenty different GBM tumor samples. Staining was observed in eight of the samples (Fig. 3c). Two types of positive staining were evident: scattered positive cells within a predominantly negative sample (5/20), or diffuse staining across many cells in the sample (3/20). Collectively, these data indicate that CD155 expression is frequently elevated in primary tumors. Since normal and cancerous tissue samples were not collected from the same patient, we cannot determine if elevated CD155 expression correlates with tumorigenesis, but speculate that such an association may exist.

### CD155/PVR is recruited to the leading edge of migrating tumor cells and colocalizes with actin and αv-integrin

**Figure 4:**
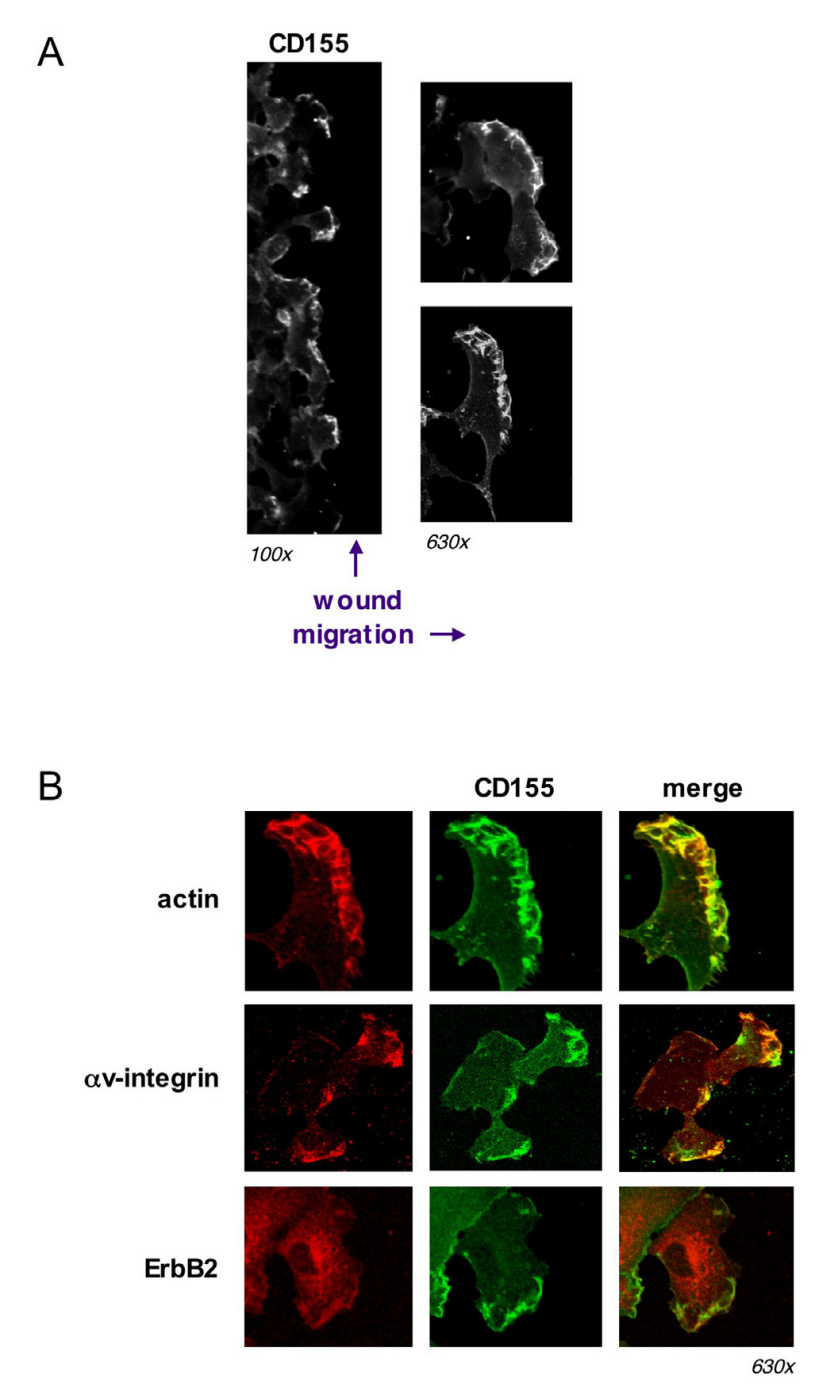
**CD155 is recruited to the leading edge of migrating cells and colocalizes with actin and αv-integrin. (a) **HT1080 cells were grown to near confluency on a matrigel substrate. A linear wound was made with a 20 g needle and cells allowed to recover for 3 h. Cells were fixed and immunostained for CD155. The majority of CD155 appeared to localize to the leading edge of migrating cells. **(b) **CD155 colocalized extensively with actin and αv-integrin at the leading edge of HT1080 cells migrating on a matrigel substrate. CD155 did not appear to colocalize with the growth-factor receptor ErbB2.

To address the role of CD155 in migrating cells, we employed a modified wound-healing assay and examined the sub-cellular localization of CD155. HT1080 cells were plated onto chambered tissue culture slides coated with a thin layer of Matrigel ECM substrate and grown to near confluence. A linear wound was made using a 20-gauge needle and the cells were allowed to recover for 3 h. Cells were fixed and immunostained to visualize CD155. CD155 was found to preferentially localize to the leading edge of cells, though some staining in trailing edges and cell-cell contacts could also be seen (Fig. 4a). In cells plated in isolation, where directionality could not be established, CD155 was consistently observed at some but not all peripheral edges of cells (data not shown). These results suggest that CD155 is recruited to the leading edge of migrating cells where it may be involved in directional motility.

Given our findings that CD155 was important for cell migration and that it localized to the leading edge of migrating cells, we asked whether CD155 might co-localize with other proteins known to be involved in motility. We used immunofluorescence and confocal microscopy to visualize CD155 along with actin and αv-integrin. CD155 colocalized extensively with actin ruffles at the leading edge of migrating cells (Fig. 4b), suggesting a potential link between CD155 and the actin cytoskeleton. CD155 also colocalized with αv-integrin, a known mediator of ECM adhesion [20], but not the epidermal growth factor receptor family member ErbB2, a mediator of growth factor signaling [21]. Thus, CD155 is associated with key players in substrate adhesion at the leading edge, and may be working in concert to mediate motility.

### CD155/PVR influences cellular morphology

**Figure 5:**
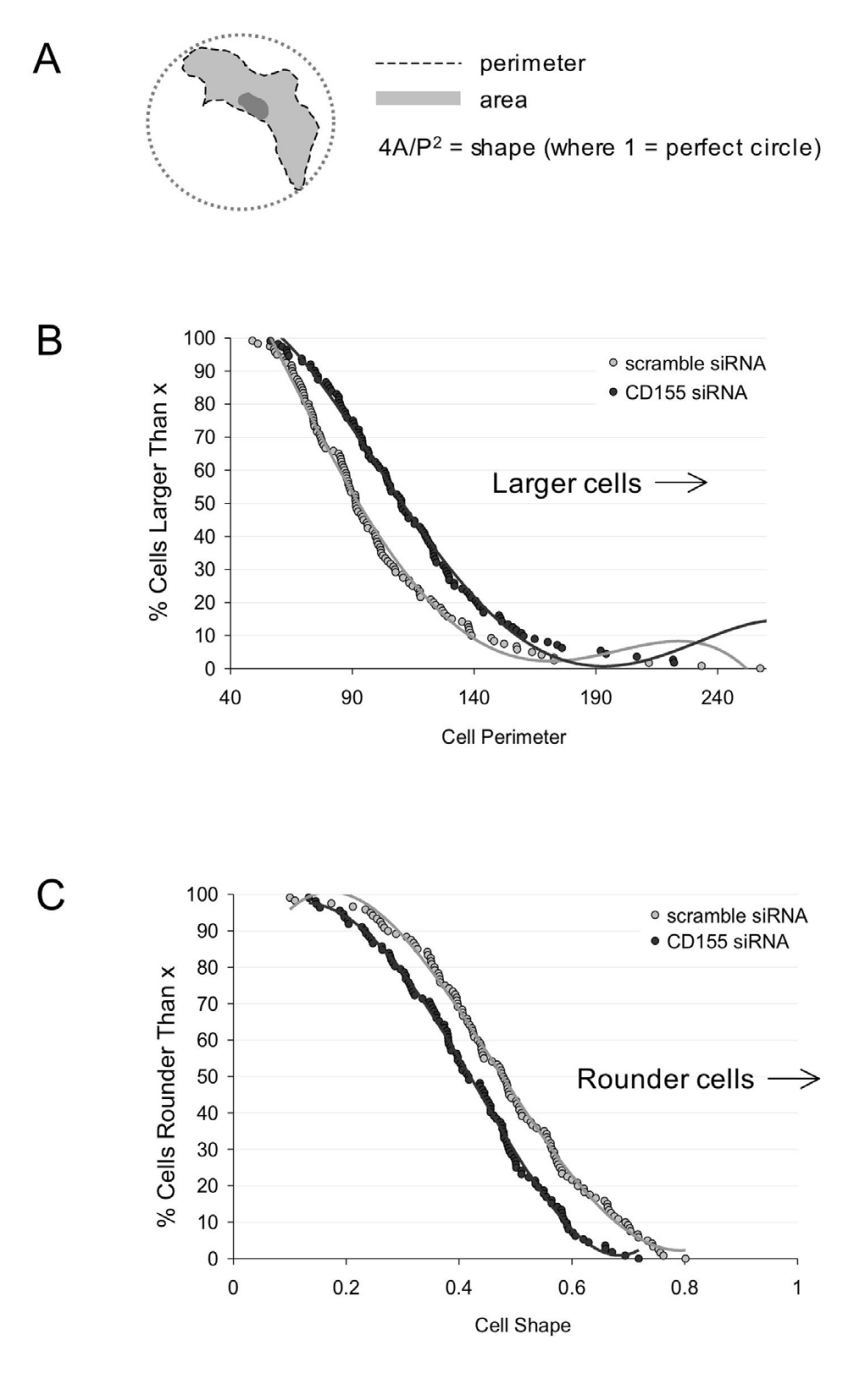
**CD155 regulates cellular morphology. **HT1080 cells were transfected with siRNA targeting CD155 or with a scrambled control duplex and plated on matrigel after 48 h. Cells were fixed and immunostained for CD155 expression before images were acquired for morphometric analysis. **(a) **Schematic showing measurement parameters. **(b) **Chang plot showing cell perimeter measurements. Perimeters values are expressed as percent of the average control perimeter. As a population, CD155 knockdown cells (dark circles) exhibited a significant increase in perimeter compared to controls (light circles). **(c) **Chang plot showing cell shape measurements. Cell shape was defined using the equation (4 × cell area) / cell perimeter^2^, where 1 indicates a perfect circle and smaller values indicate a more irregular shape. CD155 knockdown cells (dark circles) were more irregular in shape than scrambled controls (light circles). *Perimeter: *Δ *avg = +18%; shape: *Δ *avg = -13%;p < 0.001, t-test; n = 120 scramble siRNA and 112 CD155 siRNA.*

The previous section showed that CD155 localizes to the leading edge of migrating cells and colocalizes with known mediators of motility. Since cell shape changes are often associated with changes in motility and/or adhesion, we next investigated whether knockdown of CD155 by RNAi affected cellular morphology. HT1080 cells were transfected with either a CD155-specific or scrambled control siRNA (200 nM) along with a fluorescent, non-specific oligo (10 nM) to identify transfected cells. 48 hours after transfection, cells were plated at low density onto coverslips that had previously been coated with Matrigel, fixed, and immunostained for CD155 to confirm protein knockdown. Only cells containing fluorescent oligo were selected for analysis. CD155 knockdown cells had significantly larger perimeters and appeared more irregular in shape than control cells (Fig. 5). These findings suggest that CD155 has a role in cell size and shape, perhaps by regulating adhesion of cells to their substrate.

### CD155/PVR regulates migration of glioblastoma cells

**Figure 6:**
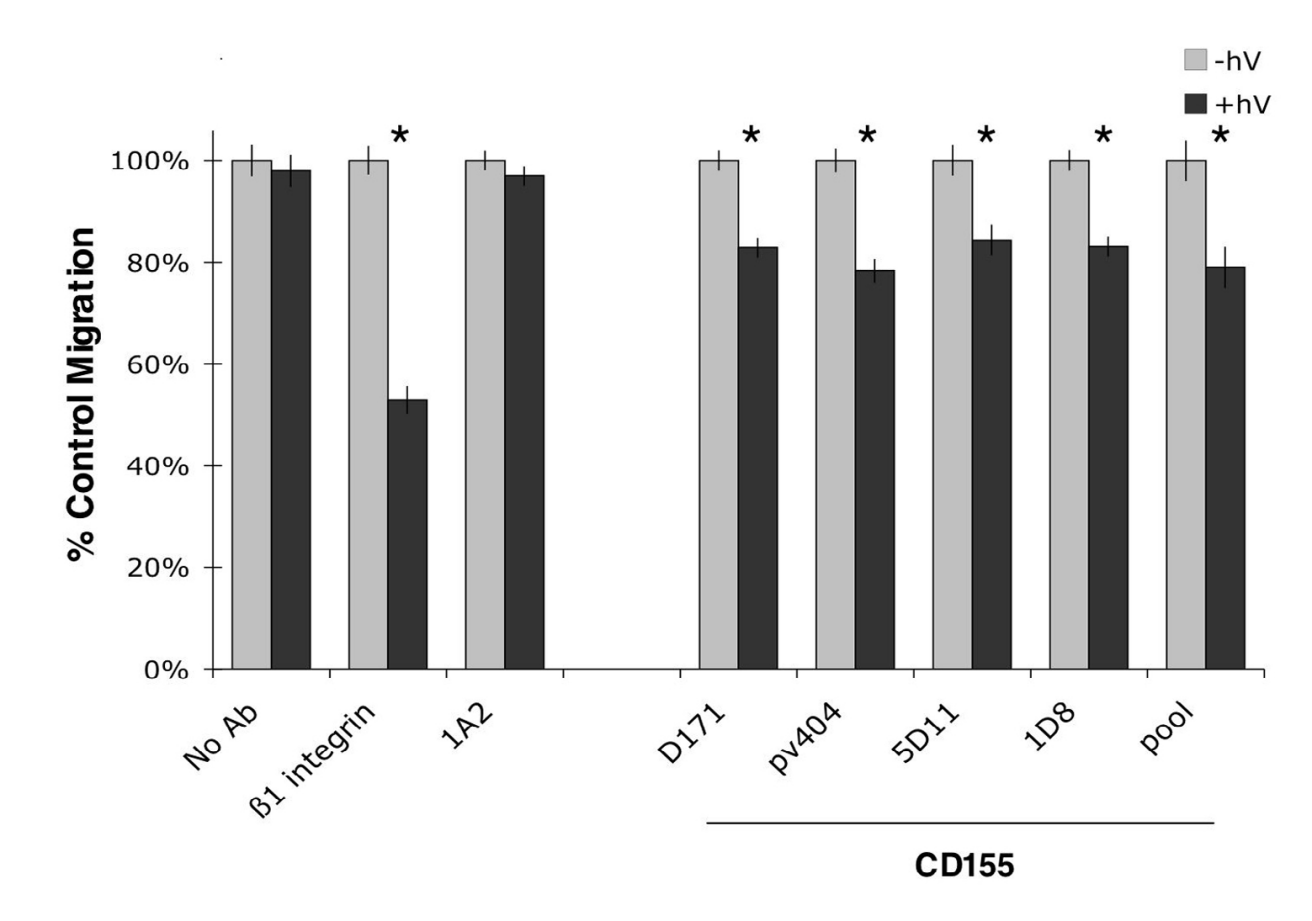
**CD155 mediates motility of GBM cells. **FALI of CD155 using multiple mAbs resulted in a significant inhibition of U87MG GBM cell transwell migration towards a serum chemoattractant (no Ab 3 ± 3%; β1-int 47 ± 3%; 1A2 (control, surface binding scFv) 3 ± 2%; CD155: D171 17 ± 2%; pv404 22 ± 2%; 5D11 16 ± 3%; IDS 17 ± 2%; CD155pool21 ± 4%; * indicates p < 0.01, t-test). FALI with CD155 mAb pools did not yield greater inhibition, suggesting maximal inactivation or a common binding site.

Our studies so far have been conducted using HT1080 fibrosarcoma cells. To assess the generality of CD155 function in cancer, we asked whether it could regulate the migration of other tumor cells *in vitro*. Our expression studies suggested that CD155 was upregulated in a subset of glioblastoma (GBM) tumors for which migratory behavior is poorly understood [3]. U87MG GBM cells were found to express high levels of CD155 (Fig. 3a). Knockdown of CD155 protein by FALI in U87MG cells resulted in a significant (16 to 22%) decrease in transwell migration towards a serum chemoattractant (p < 0.01, t-test; Fig. 6). FALI in the absence of antibody or in the presence of scFv 1A2, which binds to the surface of U87MG cells, did not alter migration. Cellular viability and proliferation were unchanged (data not shown). These results were consistently reproduced using multiple independent monoclonal antibodies targeting CD155 (D171, pv404, 5D11, 1D8). FALI with combinations of these antibodies did not enhance the inhibition of migration (Fig. 6), similar to our observations in HT1080 cells. These results demonstrate a role for CD155 in regulating migration across multiple tumor cell types.

## Discussion

We developed a functional proteomic screen to identify surface proteins involved in tumor cell invasion [16] and here have expanded it to interrogate a library of 338 single chain phage display antibodies. One of the proteins identified as a mediator of invasion was CD155, the poliovirus receptor. This work reveals a novel role for CD155 as a mediator of tumor invasion that is likely due to its function in cell migration.

Knockdown of CD155 by FALI or by RNAi resulted in impaired *in vitro *migration and a pronounced change in cellular morphology with cells becoming more elongated and irregular in shape. CD155 localizes to the leading edge of migrating tumor cells and co-localizes with actin ruffles and αv-integrin, suggesting that CD155 may act in motility and/or cell-substrate adhesion. We also observed elevated expression of CD155 in a number of different cancer cell lines and primary tumors, suggesting a link between CD155 and tumorigenesis.

The endogenous function of CD155 is not well understood. CD155 is a type I transmembrane glycoprotein first identified based on its ability to mediate the binding of poliovirus to host cells [22]. It is a member of the immunoglobulin (Ig) superfamily and belongs to a subclass that contains three Ig-like domains (V-C2-C2). This subclass includes the nectins and several nectin-like proteins including the rodent *Tage4 *gene [23]. Nectins have been implicated in organizing cell-adherens junctions through homo-and heterophillic adhesion [24,25]. While nectins bind to the actin cytoskeleton through afadin, CD155 does not [24], suggesting that CD155's cellular role is distinct from that of nectins.

Several proteins have been found to interact with CD155. The ECM protein vitronectin binds CD155 *in vitro *suggesting that CD155 may mediate cell-substrate adhesion [26]. Our findings that CD155 co-localizes with αv-integrin, a receptor for numerous ECM proteins including vitronectin, is consistent with previous reports [23] and suggests a functional role for CD155 in mediating adhesion. Activation of integrins leads to assembly of focal adhesion complexes that stabilize cellular interaction with its substrate through intracellular signaling and rearrangement of the actin cytoskeleton [27]. Our experiments showed that loss of CD155 inhibited migration and induced cell spreading. This phenotype is similar to that observed in F397-FAK fibroblasts in which focal adhesions are enhanced and cell spreading is increased [28]. We speculate that CD155 could be involved in modulating integrin/substrate interactions leading to decreased adhesion or increased turnover of focal adhesions. Another Ig-domain containing protein, CD47, has been shown to bind to αv-integrin and is present in early adhesion complexes at the leading edge of spreading melanoma and human vascular endothelial (HUVEC) cells [29]. CD47 appears to regulate integrin activation and contribute to integrin-mediated adhesion events [30]. CD155 has also been shown to interact with nectin-3 [23], the dynein motor protein Tctex-1 [31], and also to reside proximal to CD44 [32]. Future studies will address the importance of these interactions for cancer cell migration.

Our results, in which CD155 inhibition reduces migration, are also consistent with a model in which transient interactions between CD155 and ECM result in pro-migratory signals. For example, it is known that binding of integrins to ECM proteins induces pro-migratory signaling through clustering of associated kinases [33]. CD155 itself may transduce signals when bound to ECM proteins or could be involved in the clustering of larger complexes. During preparation of this manuscript, Oda et al. reported that crosslinking of exogenously expressed CD155 resulted in tyrosine phosphorylation of its cytoplasmic tail, and concurrent reductions in focal adhesion kinase (FAK) and paxillin phosphorylation in NIH3T3 mouse fibroblasts [34]. Crosslinking of CD155 also resulted in decreased adhesion to fibronectin, a reduction in the number of focal adhesions, and an increase in migration [34]. It is possible that overexpression of CD155 in cancer cells drives dimerization of CD155 in the absence of ligand, resulting in decreased adhesion and increased migration as well as other signaling events.

CD155 expression has been reported widely to be restricted to primates [35,36], but recent work suggests that Tage4 may be a rodent ortholog [37,38]. At the amino acid level, Tage4 shares only 42% homology with CD155 [38] and rodents are not susceptible to polio virus infection [22,39]. However, Tage4 shares the extracellular structure of CD155 and the two genes reside in syntenic chromosomal regions [38]. Emerging data suggest functional similarities between the two proteins. Tage4 has been shown to bind to both nectin-3 and vitronectin [37,38] and also appears to colocalize with αvβ3-integrin [37]. Recently, Tage4 has been implicated in cell migration [37]. Overexpression of Tage4 led to increased migration of murine L cells in a serum- and integrin-dependent manner while Tage4 mutants inhibited motility [37]. Furthermore, V12Ras-transformed NIH3T3 cells, which form tumor nodules in the lungs of nude mice, were found to express elevated levels of Tage4 and expression of a dominant-negative Tage4 inhibited the ability of these cells to form nodules [37]. Due to low sequence conservation and the lack of functional data for CD155, it has been difficult to resolve whether Tage4 and CD155 are true orthologs. Our identification of a role for CD155 in tumor cell migration supports the idea that these proteins are functionally related. Thus, it will be interesting to compare the two proteins in future studies in order to better define the mechanism of action for CD155 both in normal cells and in cancer states.

We observed high levels of CD155 protein expression in a subset of several different types of primary tumor. Previously, expression of the CD155 gene had been reported to be upregulated in colon cancer [40] and possibly glioblastoma [19]. Here we have extended that observation at the protein level to several additional tumor types, including cancers of the prostate, kidney, pancreas, lung, ovary, breast, and brain, suggesting a much broader role for CD155 in tumorigenesis. A search of the EST and SAGE library databases maintained by the Cancer Genome Anatomy Project (CGAP; ) using the unique identifier AACCACCCAG supports the idea that expression of the CD155 gene may be elevated in several tumor types including colon, brain, kidney, pancreas, lung, and stomach (Table 1). Our finding that CD155 is involved in tumor cell migration in fibrosarcoma and glioblastoma cells implicates CD155 as a mediator of metastasis and dispersal. The selective expression of CD155 in tumors compared to normal tissue further supports the idea that targeted inhibition of CD155 could serve as a useful therapeutic approach to limit the spread of tumor cells *in vivo*. Very recently, Ochiai et al. reported that CD155 expression is upregulated in several breast cancer cell lines and primary breast tumors and demonstrated that an oncolytic poliovirus recombinant delivering a toxic payload could selectively kill breast cancer cells [41]. Our findings suggest that such a therapeutic approach could also have value in treating other cancer types. Since elevated expression of CD155 was detected in a subset of samples from the examined tumor types, it is possible that CD155 expression may represent a late-stage event in tumorigenesis. It is also possible that CD155 could lie in one of several different oncogenic pathways. Further research is necessary to determine if and how CD155 contributes to cancer progression *in vivo*.

This work extends the utility of our FALI-based functional screening approach in identifying proteins with a role in tumor cell invasion. Our previous screen identified an extracellular role for Hsp90 in regulating tumor cell invasion through regulation of MMP2 activity [16]. In contrast, this screen has identified a role for CD155 in regulating tumor cell motility. Thus, our approach can yield novel mediators of tumor cell invasion that are involved either directly in invasion or more generally in cell migration. Future applications of this technology will likely yield additional validated targets and open new avenues for research.

## Conclusions

In summary, we have applied a novel, functional proteomic approach to identify proteins that mediate tumor cell invasion and have identified a novel role for CD155 in regulating cancer cell invasion and migration. We suggest that CD155 may control migration by regulating cell-substrate adhesion. We have further shown that CD155 is commonly expressed at high levels in primary tumors and speculate that it may contribute directly to tumorigenesis by enhancing cancer cell migration *in vivo*.

## Competing interests

KS, BE, JS, MS, JR, DL: none declared

CZ, CT, CU, LI, DJ: received funding (research support or salaries) from Xerion Pharmaceuticals, AG.

## Authors' contributions

KS contributed to study design, data interpretation, carried out the migration experiments with FALI and RNAi and the characterization of CD155 expression, and drafted the manuscript. BE contributed to study design, data interpretation, carried out the invasion screen and morphology experiments, and helped revise the manuscript. JS contributed to the invasion screen. CZ and CU generated and characterized the scFv library. CT performed the IP and mass spec to identify CD155. MS helped with the morphology experiments. JR and DL performed the GBM tissue staining and analysis and provided helpful discussion. LI and DJ contributed to study design, data interpretation, and editing of the manuscript.

## Pre-publication history

The pre-publication history for this paper can be accessed here:



## Figures and Tables

**Table 1:** Results of CGAP EST and SAGE library search for CD155

	**EST data ***(tags/200,000)*	
**Tissue**	**Normal**	**Cancer**
Lung	4	37
Genitourinary	0	35
Head and neck	0	27
Stomach	0	23
Colon	0	22
Pancreas	0	20
Kidney	3	17
Bone	0	14
Cervix	0	10

	**SAGE data ***(pos libraries/ total libraries)*	
**Tissue**	**Normal**	**Cancer**

Brain	0/8	21/63
Lung	0/1	2/3
Pancreas	0/1	2/2
Kidney	0/1	1/1
Thyroid	0/1	1/1
